# Atypical Parkinsonism as a Manifestation of Central Nervous System Lymphoma

**DOI:** 10.7759/cureus.104217

**Published:** 2026-02-25

**Authors:** Sara Gomes, Sofia Lopes, Stefanie Moreira, José Manuel Araújo, Eduardo Freitas

**Affiliations:** 1 Department of Neurology, Local Health Unit of Braga, Braga, PRT

**Keywords:** atypical manifestation, central nervous system lymphoma, imaging features, parkinsonism, progressive supranuclear palsy

## Abstract

Primary central nervous system (CNS) lymphoma is an uncommon form of non-Hodgkin lymphoma with a wide range of presentations that can mimic various neurological diseases, including neurodegenerative disorders.

We report the case of a 74-year-old man admitted with a seven-month history of progressive cognitive decline and gait disturbances. On examination, he presented cognitive dysfunction, impaired ocular movements, right-predominant parkinsonism, and postural instability. Initial investigations, including imaging studies, electroencephalography, cerebrospinal fluid analysis, and laboratory tests, did not reveal relevant abnormalities, leading to a working diagnosis of probable progressive supranuclear palsy. The patient’s condition continued to worsen, and a repeat MRI was performed, revealing findings suggestive of lymphoma. A biopsy confirmed the diagnosis, and corticosteroid therapy was initiated. The patient was subsequently referred to hematology.

Although parkinsonism is rarely an initial manifestation of primary CNS lymphoma, the duration and rapid progression of symptoms warranted inpatient investigation, enabling the correct diagnosis and timely initiation of treatment of a potentially curable malignancy.

## Introduction

Primary central nervous system (CNS) lymphoma is an uncommon form of non-Hodgkin lymphoma, with diffuse large B-cell lymphoma being the most common subtype [1]. It most frequently involves the brain parenchyma, but it can also affect the leptomeninges, spinal cord, or retina [2].

Immunosuppression is the main risk factor, particularly in patients with human immunodeficiency virus (HIV) or those who have received organ transplants. However, its incidence has been increasing in immunocompetent individuals over 65 years of age [1,2].

The diagnosis of primary CNS lymphoma can be challenging due to its wide range of presentations, including focal neurological deficits, seizures, and neuropsychiatric symptoms. It can mimic several neurological diseases, including neurodegenerative disorders, particularly atypical parkinsonian syndromes [2,3].

Although contrast-enhanced MRI is essential, lymphoma presents with considerable radiological heterogeneity; therefore, definitive diagnosis relies on histological confirmation obtained by stereotactic biopsy. CSF samples should also be collected for diagnosis and staging [2,4,5].

This case describes a patient presenting with ocular abnormalities, postural instability, akinesia, and cognitive dysfunction, mimicking progressive supranuclear palsy (PSP), with symptoms ultimately found to be secondary to lymphoma. This highlights primary CNS lymphoma as a treatable mimic of PSP and the diagnostic value of repeat MRI in rapidly progressive parkinsonism.

## Case presentation

A 74-year-old man with a medical history of arterial hypertension, type 2 diabetes mellitus, dyslipidemia, and alcohol abuse was admitted for evaluation of a seven-month history of progressive cognitive decline and gait disturbances associated with recurrent falls. On examination, he exhibited psychomotor slowing, with an objectively measured cognitive decline, with temporal disorientation and difficulties in calculation, recall, and drawing. Additional findings included hypomimia and hypophonia; impaired ocular movements with limited downgaze and hypometric saccades; right-predominant bradykinesia with a subtle, low-frequency resting tremor; and a broad-based, short-stepped gait with marked postural instability.

**Figure 1:**
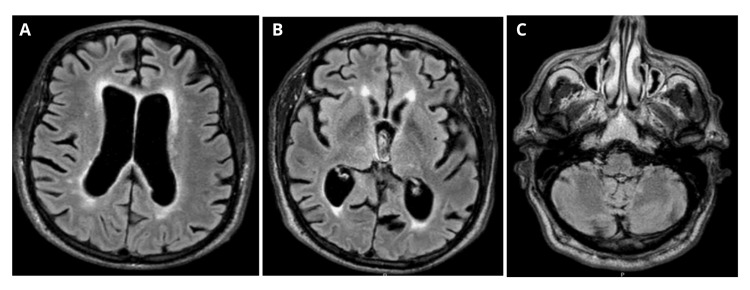
Brain MRI showing subcortical atrophy and white matter lesions due to small-vessel disease in T2/FLAIR (A-C) FLAIR: Fluid-attenuated inversion recovery

He underwent investigation with brain and spinal MRI, which demonstrated subcortical atrophy, white matter lesions due to small-vessel disease (Figure 1), lumbar canal stenosis, and foraminal narrowing at L4-L5, with a deferred surgical indication by neurosurgery. EEG was unremarkable. CSF analysis revealed 4 cells/µL, protein 0.62 g/L and normal glucose. Alzheimer’s disease biomarkers showed reduced beta-amyloid (Aβ42). The autoimmune encephalitis panel was negative.

Given the duration of symptoms and the suspicion of atypical parkinsonism, autoimmune encephalitis, or another paraneoplastic syndrome, a paraneoplastic workup was performed, including a comprehensive laboratory panel, which showed no relevant changes. Contrast-enhanced CT of the thorax, abdomen, and pelvis, together with whole-body FDG-positron emission tomography (PET), raised suspicion of an infiltrative colonic lesion. Subsequent endoscopic evaluation with biopsy, however, was negative for neoplasia.

Based on the clinical presentation with ocular abnormalities, postural instability, akinesia, and cognitive dysfunction and diagnostic findings, a working diagnosis of probable PSP was established, and a trial of levodopa was initiated and titrated up to 400 mg per day, resulting in slight improvement in bradykinesia and postural stability.

**Figure 2:**
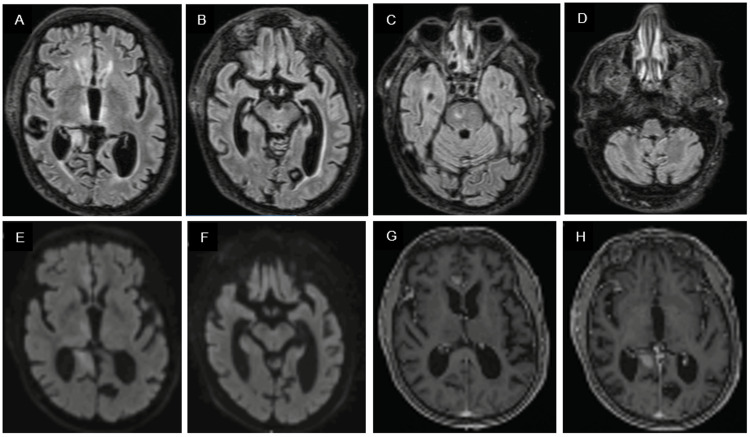
Brain MRI (two weeks later) revealing T2/FLAIR hyperintense lesions involving the thalami, midbrain, bilateral cingulate gyri, frontobasal and right anterotemporal basal regions, as well as the genu of the corpus callosum (A-D), with diffusion restriction on DWI (E and F) and contrast enhancement on post-contrast T1-weighted images (G and H). FLAIR: Fluid-attenuated inversion recovery

Over the following two weeks, the patient’s condition progressively worsened, with increasing confusion and somnolence. A new MRI was obtained, revealing T2/fluid-attenuated inversion recovery (FLAIR) hyperintense lesions in the thalami, midbrain, bilateral cingulate gyri, fronto-basal and right anterotemporal-basal regions, and the genu of the corpus callosum, with diffusion restriction and contrast enhancement, suggestive of lymphoma (Figure 2).

Based on these findings, lumbar puncture was repeated, with flow cytometry and cytologic analysis for neoplastic cells, both of which were negative.

The case was discussed with the neurosurgery team, who decided to perform a biopsy. Histology confirmed the diagnosis of diffuse large B-cell lymphoma.

Primary CNS lymphoma was confirmed as the final diagnosis in the absence of systemic involvement. Corticosteroid therapy was initiated, with clinical stabilization, and the patient was referred to hematology for follow-up and palliative care planning due to his frailty.

## Discussion

Primary CNS lymphoma can present with clinical features similar to PSP when it infiltrates brain regions critical for motor, cognitive, and oculomotor function, particularly the basal ganglia, thalamus, and midbrain. This anatomical overlap can result in a constellation of symptoms including cognitive decline, postural instability, supranuclear gaze palsy, and akinesia: hallmarks of PSP [6,7].

The underlying mechanism is direct lymphomatous infiltration or disruption of neural circuits in these regions, especially when lesions are bilateral and involve the globus pallidus or midbrain [6,8].

Unlike classic PSP, which is a tauopathy with insidious onset and slow progression, CNS lymphoma typically evolves more rapidly and may be accompanied by additional features such as apraxia and atypical imaging findings [3].

In the present case, the initial manifestations consisted of cognitive decline and postural instability, later progressing to vertical gaze palsy and akinesia. Given the relatively short time course, paraneoplastic causes needed to be excluded through an extensive workup, which initially did not reveal any malignancy. The diagnosis of PSP was therefore considered, based on the presence of the four cardinal clinical features and an apparently gradual clinical progression. Notably, the initial brain MRI was unremarkable; however, repeat imaging performed two weeks later revealed infiltrative lesions consistent with lymphoma, underscoring the critical importance of repeating neuroimaging in the setting of rapid clinical deterioration.

Recognizing primary CNS lymphoma in this context is crucial, as it represents a potentially curable malignancy with appropriate treatment, in contrast to PSP, which remains incurable and relentlessly progressive. 

Characteristic imaging features of this lymphoma subtype included iso- to hypointense T1-weighted signal, T2/FLAIR hyperintensity, homogeneous contrast enhancement, and marked diffusion restriction, with possible involvement of the basal ganglia, thalamus, and midbrain [2,4,5].

To guide treatment, patients are first stratified as fit or unfit for chemotherapy, and subsequently as eligible or ineligible for autologous stem cell transplantation, with the regimen consisting of an induction phase followed by a consolidation phase. The cornerstone of treatment is chemotherapy protocols, with or without the addition of radiotherapy, immunotherapy, and corticosteroids. Despite therapeutic advances, more than half of the patients experience relapse [2,5].

Long-term management includes clinical and imaging assessments every three months initially, and subsequently every six months, to evaluate treatment response, symptom improvement, and complications. Patients who are unfit for treatment due to age, limited tolerance to intensive therapies, performance status, organ function, comorbidities, or frailty may be offered palliative care, as in the case presented [5].

## Conclusions

Parkinsonism is rarely an initial manifestation of primary CNS lymphoma, making the diagnosis challenging. In this case, the duration and rapid progression of symptoms prompted inpatient investigation. Early repeat imaging allowed accurate identification of the underlying disease, guided biopsy, and timely initiation of treatment.
